# An Artificial Intelligence Chatbot for Young People’s Sexual and Reproductive Health in India (SnehAI): Instrumental Case Study

**DOI:** 10.2196/29969

**Published:** 2022-01-03

**Authors:** Hua Wang, Sneha Gupta, Arvind Singhal, Poonam Muttreja, Sanghamitra Singh, Poorva Sharma, Alice Piterova

**Affiliations:** 1 Department of Communication University at Buffalo, The State University of New York Buffalo, NY United States; 2 Department of Communication The University of Texas at El Paso El Paso, TX United States; 3 School of Business and Social Sciences Inland University of Applied Sciences Elverum Norway; 4 Population Foundation of India New Delhi India; 5 AI for Good UK London United Kingdom

**Keywords:** artificial intelligence, chatbot, Facebook, affordance, sex education, sexual and reproductive health, contraception, case study, young people, India, transmedia, mobile apps, mobile health, technology design, user engagement, digital health, mobile phone

## Abstract

**Background:**

Leveraging artificial intelligence (AI)–driven apps for health education and promotion can help in the accomplishment of several United Nations sustainable development goals. SnehAI, developed by the Population Foundation of India, is the first Hinglish (Hindi + English) AI chatbot, deliberately designed for social and behavioral changes in India. It provides a private, nonjudgmental, and safe space to spur conversations about taboo topics (such as safe sex and family planning) and offers accurate, relatable, and trustworthy information and resources.

**Objective:**

This study aims to use the Gibson theory of affordances to examine SnehAI and offer scholarly guidance on how AI chatbots can be used to educate adolescents and young adults, promote sexual and reproductive health, and advocate for the health entitlements of women and girls in India.

**Methods:**

We adopted an instrumental case study approach that allowed us to explore SnehAI from the perspectives of technology design, program implementation, and user engagement. We also used a mix of qualitative insights and quantitative analytics data to triangulate our findings.

**Results:**

SnehAI demonstrated strong evidence across fifteen functional affordances: accessibility, multimodality, nonlinearity, compellability, queriosity, editability, visibility, interactivity, customizability, trackability, scalability, glocalizability, inclusivity, connectivity, and actionability. SnehAI also effectively engaged its users, especially young men, with 8.2 million messages exchanged across a 5-month period. Almost half of the incoming user messages were texts of deeply personal questions and concerns about sexual and reproductive health, as well as allied topics. Overall, SnehAI successfully presented itself as a trusted friend and mentor; the curated content was both entertaining and educational, and the natural language processing system worked effectively to personalize the chatbot response and optimize user experience.

**Conclusions:**

SnehAI represents an innovative, engaging, and educational intervention that enables vulnerable and hard-to-reach population groups to talk and learn about sensitive and important issues. SnehAI is a powerful testimonial of the vital potential that lies in AI technologies for social good.

## Introduction

### Background

This paper presents rich insights from an instrumental case study of an innovative chatbot in India called SnehAI, which was purposefully conceptualized and designed by the Population Foundation of India to educate and inspire adolescents and young adults to live healthy lives, promote sexual and reproductive health (SRH), and advocate for the health and well-being of women and girls. The SnehAI chatbot aims to provide a safe space for Indian youth to have conversations about SRH, dispel sex-related myths and taboos, offer accurate information about safe sex and contraceptive choices, and address mental health concerns. With a population of approximately 1.4 billion, India accounts for about 18% of all people on the planet [1], with half of this population being under the age of 25 years [2]. Despite stated policy commitments and significant strides made in recent years, the informational needs of adolescents and youth are poorly met, quality education about SRH is highly limited, contraceptive practices are heavily skewed toward female sterilization, and unsafe abortions are rampant [3-6].

In particular, young people in India have limited awareness of contraception and sexually transmitted infections; their knowledge base consists of inaccurate information; and their family life education is highly insufficient [7]. Although the government-endorsed national adolescent health program Rashtriya Kishor Swasthya Karyakram (RKSK) has included SRH as part of its mandate since 2014, direct contact with frontline health workers, even by married young women, was extremely low. Contact with unmarried youth and use of SRH services at adolescent-friendly health clinics are almost completely amiss [8]. Uncomfortable and embarrassed to ask, young people in India have increasingly referred to web-based platforms to look for answers to SRH questions and have garnered misleading or incorrect information [9,10]. In a day and age when mobile services and social media are proliferating in India, this is unfortunate. At the beginning of 2020, India boasted of around 1.1 billion mobile phone connections, covering 78% of the population [11]. With attractive pricing from India-based telecom giants, such as Jio, internet penetration and social media use through mobile networks are rapidly growing [11,12]. Facebook (Meta Platforms) is an obvious leader in the social media space in India, with 320 million users [13]. With this massive expansion of information infrastructure comprising wireless networks, digital technologies, and social media, Indian youths, both in urban and rural areas, are increasingly being plugged into this technology web.

One technology within the realm of social media that has experienced a rapid rise in different industries is chatbots [14]. Chatbots are automated nonhuman agents that engage in conversations with human actors [14]. By design, the user experience in a chatbot strives to be pleasant, as it mimics a scenario in which 2 humans are talking with each other. A chatbot responds by accessing information stored in large digital data repositories. Chatbots quickly sieve what is relevant and convert programing codes into expressions that humans can understand. Although chatbots are often text based, their capabilities have exploded since the pioneering program ELIZA [15], especially in their increased sophistication and accuracy in understanding natural language using artificial intelligence (AI) technologies [16,17]. With AI, chatbots create a dynamic library of answers, building on their existing database with each conversation that occurs. This process of machine learning makes their deployment immensely valuable for disseminating information. Not surprisingly, AI chatbot health apps are burgeoning [18-21], and they have unique advantages for addressing sensitive and taboo issues such as SRH [22]. However, to date, AI chatbot initiatives in low-income countries are scarce. Furthermore, for chatbot projects that do get underway, systematic documentation and assessment are needed to generate new knowledge for the greater public good [23].

Winner of the 2020 eNGO Challenge Award in Digital Tools and Empowerment [24], SnehAI represents the first Hinglish (Hindi + English) AI chatbot that is cocreated *with* and *for* young people, especially those from vulnerable sections of society, to deliberately facilitate communication about SRH topics and promote social and behavioral change [25,26]. It benefits the target population in many unique ways, but most importantly, it fills a gap that exists in the information around SRH. In a world where digital technologies for underdeveloped markets fail to consider consumers’ hedonic needs, such as play, romance, and entertainment, SnehAI provides an unusual repository of educational knowledge in an entertaining and engaging container, commonly referred to as *entertainment-education* or *edutainment* [27,28]. Recent anthropological studies have shown that digital natives, irrespective of their socioeconomic status, tend to be attracted to entertainment and storytelling when consuming social media content [23]. Thus, to spread crucial health information in diverse populations, the content of social media platforms needs to engage users. Therefore, the SnehAI chatbot in India offers a terrific opportunity for systematic investigation.

We focused our investigation on the different ways that SnehAI enables SRH information sharing and user engagement. First, we presented our guiding framework, the theory of affordances. Our literature review highlights both foundational arguments and affordances of relevant technologies. We then described our methodological approach, an instrumental case study that harvests the unique contributions of SnehAI. Our findings are organized by our theoretically derived research questions: first, on the functional affordances of SnehAI, and second, on the user engagement patterns of SnehAI. We concluded with a discussion of these major findings, raising implications for theory and practice, and set some directions for future endeavors.

### The Gibson Theory of Affordances

The verb to *afford* means to make available, to provide, or to offer. The noun *affordance* did not exist until ecologic psychologist Gibson [29] introduced his theory of affordances. He argued that animals (including humans) and their living environments are one world, and we cannot understand it fully if we treat them as separate entities. Essentially, we are all created by the world we live in, and our behaviors are shaped by our environment [30]. The relational dynamics between us and our environment can enable or prohibit certain action possibilities [30-32]. Presently, a smartphone can allow us to make calls, send messages, browse the web, and guide us to locations; however, without a charged battery or a correct password, nothing can be done. Gibson [30] also pointed out that affordances may easily imply a positive connotation; however, it is important to remember that they can also be negative. Certain possibilities may be enabling whereas others are constraining [31]. Presently, a mass email can easily reach several people with a click of the *send* button, but if infected, it can also spread a computer virus across the world in no time. Moreover, Gibson [30] emphasized that the affordances of an environment exist permanently—some may be latent that require discovery, but most should be directly perceivable without extra effort of learning. Affordances invite concrete behaviors, which, in turn, can afford other behaviors [30] and, in some cases, provide agency to seek value and meaning [33,34]. Most young people can figure out how to navigate various social networking sites rather intuitively; and given that their behaviors are observable to others, this can trigger additional web-based behaviors.

### Functional Affordances of Technologies

Over the past 4 decades, the theory of affordances has deeply influenced the design of everyday objects and user experiences of human–computer interaction [35-37]. Affordances have been used as a high-level theoretical framework to understand the internet [38,39], mobile and social media [40,41], and digital apps for eHealth [42-44]. As SnehAI represents an AI-driven chatbot accessible through the Facebook Messenger mobile app, our literature review focused on discussions regarding the affordances of all relevant technologies and those pertaining to health promotion and education to set the conceptual foundation for our analysis.

As the internet proliferated, reviews about its affordances emphasized its accessibility through broadband networks. Increasingly available anywhere, anytime, and in multiple media modalities (eg, text, audio, and visual), the internet embodied the novel nonlinearity of user experience beyond time and space enabled by hypertexts and search engines [38,39]. Key affordances of mobile devices include portability of smartphones, information exchange with multiple others across multiple channels with increased frequency and directness, surveillance risks tied to locational presence, and multimedia content production and sharing [40]. The key affordances of social media platforms include the permanent nature of web-based expressions that are digitally recorded and archived; the possibility for users to re-edit and recraft their messages to manage their self-presentation; the possibility for the digital content to be searched, replicated, and disseminated to a mass audience; and the increased visibility of users’ personal profiles, social connections, and web-based behaviors [41,45,46].

Although AI and chatbots have been around for decades, they have gained in currency with recent developments in cloud computing and machine learning to leverage big data [17,47]. The key affordances of AI-driven chatbots include receiving messages such as real-time status updates and aggregated information from different sources; setting options for automated reminders, nudges, and other triggers for user engagement; enabling users to query information based on personal interests; and enriching messages by having the content processed and enhanced with hyperlinks, emojis, graphics interchange formats (GIFs), and other audio-visual effects [17]. Although exploratory studies of AI-driven chatbots are scarce, their promise to address, circumvent, and overcome the taboo nature of SRH and engage young people is unmistakable [22,47].

### Research Gaps and Questions

For the most part, scholars and practitioners agree on the *existence* of affordances as specific *functions* of materials or artifacts (in our case, technologies) and the necessary *recognition* of such affordances for their *realization* to take place [17,30,44]. However, there were several gaps. First, many phrases, such as *social affordances*, *cognitive affordances*, *emotional affordances*, and *therapeutic affordances*, have become popular in the literature that are in fact inferences or implications of the functional affordances [39,42,43]. Therefore, any analysis of affordances should fundamentally be anchored to technological functionalities. The categorization of social, cognitive, emotional, and therapeutic affordances should be secondary. Making such conceptual distinctions is important.

Second, most of the literature on affordances has a positive bias and is most often limited to their potential for achieving personal goals or public good without considering concerns or risks. Painting a rosy picture of a rapidly changing media landscape is dangerous. Similar to all previous information and communication technologies, smart and connected mobile devices and social media platforms work like a double-edged sword. It is our ethical obligation, in this digital and network society, to reduce the prejudice of technological determinism [31] and make deliberate efforts to address and account for the negative affordances that can cause detrimental effects [48].

Third, AI chatbot design and research are still in their infancy [14]. As technologies advance to highly interactive user interface with realistic digital representations, it is unclear how their connection to certain contextual information, such as a background story, will affect user appeal and engagement [16,49-51]. Such technological advantages tend to serve privileged populations better and can implicitly embody prejudices against those who are vulnerable and marginalized [52].

Finally, although all the technological artifacts are embedded in some kind of regulatory system, be it legal or social, the role of these regulatory systems is rarely included in the discussion of affordances. Although we focus on what algorisms would enable or prohibit digital citizens from doing (or not doing) with their smart devices, affordances of AI operate in the space of public discourses that can affect individual autonomy and privacy [53,54].

On the basis of the above literature review and identified gaps, we investigated the SnehAI chatbot as an AI-driven conversational agent and a digital tool for sex education and communication about SRH, nested in the Facebook Messenger mobile app. By applying the theory of affordances as a high-level conceptual framework, by connecting and synthesizing all relevant affordances from the literature, and by analyzing both qualitative insights and unobtrusive quantitative behavioral data, we answered the following research questions:

1. Research question 1: What are the functional affordances of SnehAI for promoting conversations around SRH?
2. Research question 2: How are users engaging with SnehAI in their conversations?

## Methods

### Study Context

SnehAI was first launched in April 2019. Although its current version is a stand-alone AI chatbot, its original idea and predecessor were deeply rooted in the Population Foundation of India’s innovative initiative called *Main Kuch Bhi Kar Sakti Hoon* (*I, A Woman, Can Achieve Anything*). This initiative used a 360° approach, especially the *transmedia edutainment* social and behavior change communication strategy [55,56], coordinated across multiple media platforms to challenge deeply entrenched regressive gender norms and to advocate for women’s empowerment. Powerful stories were told through the dramatic journey and positive role modeling of protagonist Dr Sneha, who leaves behind a lucrative medical practice in Mumbai and returns to her home village Pratappur after her sister’s death from a forced abortion. She then stays on to tackle multiple social ills—child marriage, sex selection in favor of male offspring, violence against women and girls, and many other manifestations of gender inequality [57].

From March 2014 to September 2019, 3 seasons of *Main Kuch Bhi Kar Sakti Hoon* were broadcast on the Indian national television network Doordarshan, hundreds of All India Radio stations, and the Mobile Vaani community [58,59]. *Main Kuch Bhi Kar Sakti Hoon* was the first to use an interactive voice response system for large-scale real-time audience engagement, creating a *voicebook* as a transmedia extension that allowed millions to interact with curated content, answer questions, and share personal opinions and actions inspired by the characters in the program [58]. Several miniseries were created out of the television content, extending the storyline on *Main Kuch Bhi Kar Sakti Hoon*’s Facebook page and YouTube channel [57,60]. In particular, its Facebook page was popular among young people and male users according to Insights Analytics in October 2019 (Multimedia Appendix 1).

In April 2019, during season 3, *Main Kuch Bhi Kar Sakti Hoon* launched a new transmedia extension through Facebook Messenger: a chatbot named after Dr Sneha, *SnehAI* (for Sneha AI). SnehAI was created by the Population Foundation of India in close partnership with the UK-based innovative technology company AI for Good. This AI chatbot was purposefully designed to extend Dr Sneha’s media personality as *a trusted friend* and open a safe, nonjudgmental, and private channel to engage the audience (especially young people) in India to talk and learn about SRH. SnehAI version 1.0 used a built-in decision tree with predetermined rules and quick replies to help users navigate through the visual menus [25,26]. It was cocreated with invaluable inputs from 84 adolescents and 19 adults to ensure a friendly tone of voice with familiar colloquial expressions that, in fact, SnehAI became the world’s first ever Hinglish AI chatbot developed for social and behavioral change [25,26]. It was promoted through *Main Kuch Bhi Kar Sakti Hoon*’s website, interactive voice response system, Facebook page, and other channels. Building on previous efforts, this paper focuses on the current version of the AI chatbot, SnehAI version 2.0.

### SnehAI Chatbot

**Figure 1:**
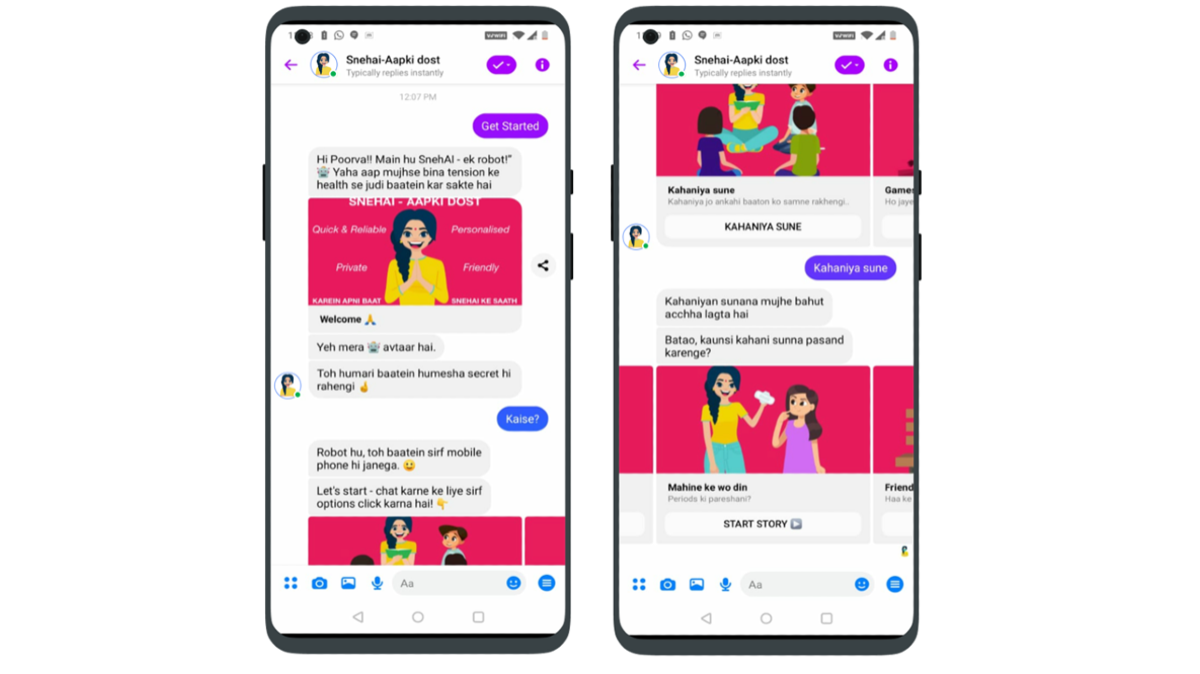
SnehAI chatbot user interface on Facebook Messenger.

In April 2020, SnehAI (version 2.0) was launched with a natural language processing (NLP) platform and better content flow to make SnehAI more intelligent in her conversations with users [26]. It is required that an individual be a user of Facebook Messenger to chat with SnehAI (Figure 1).

**Figure 2:**
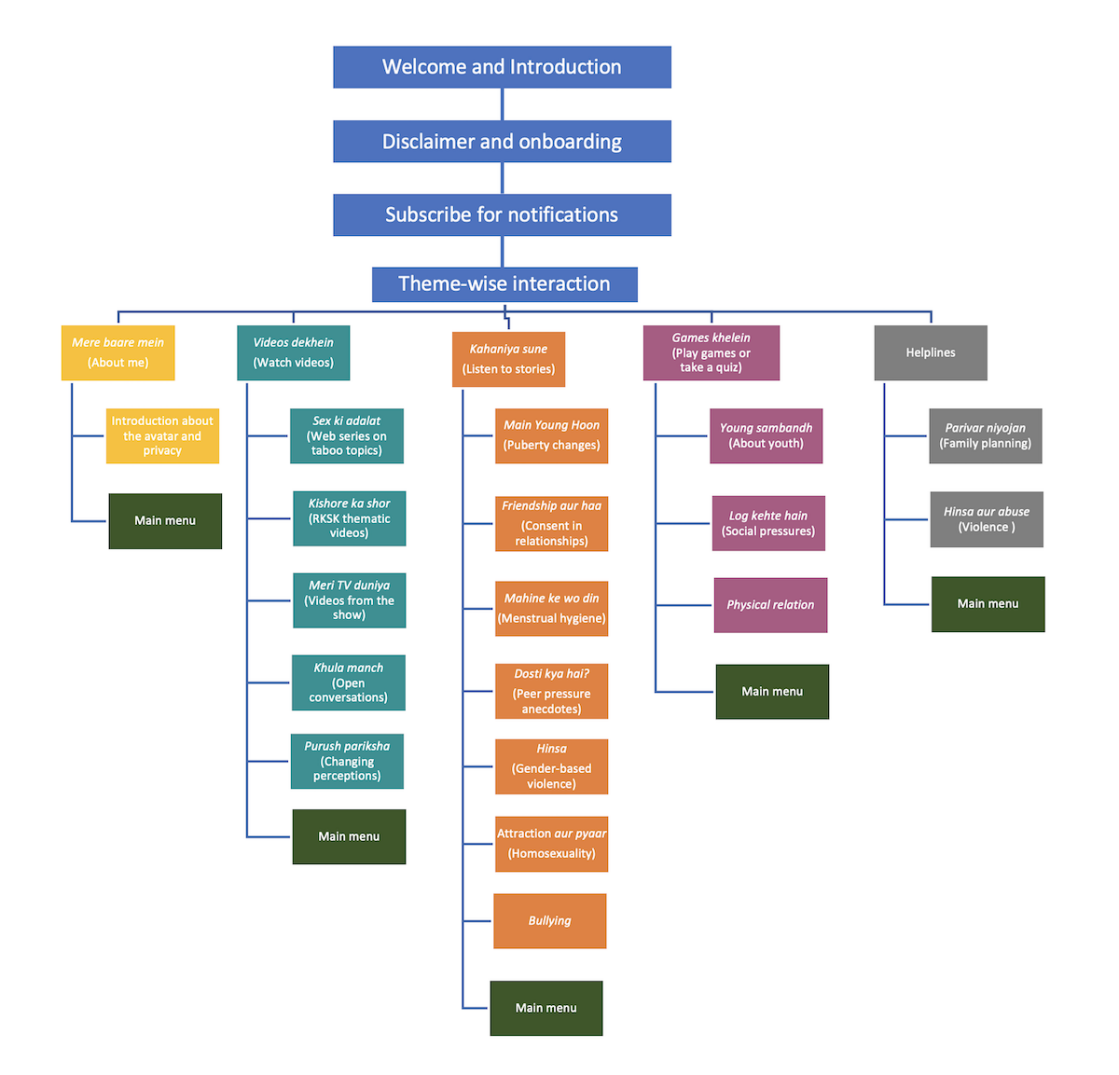
SnehAI chatbot content flowchart. RKSK: Rashtriya Kishor Swasthya Karyakram.

The conversation between a user and SnehAI starts with a warm welcoming message. The chatbot introduces itself as Dr Sneha. She mentions that she works in a hospital and is involved in other development projects in the village. After introduction, she asks the user to choose one of the available options to ensure that the user understands that he or she is interacting with a chatbot rather than a human being. After that, the users are invited to subscribe to notifications from SnehAI. Then they can select and interact with a range of content presented on the main menu. Depending on their choices, the information may be displayed as hyperlinked texts, images, or short videos. Furthermore, these theme-wise interactions are organized into five content categories (Figure 2):

1. *Mere baare mein* (About me): this is an introduction to the app. SnehAI is introduced as an avatar, and users are also given the option to review the privacy policy. In the chatbot, this option appears at the end, as the goal is to promote health-related communication.
2. *Videos dekhein* (Watch videos): this option allows the user to watch short videos from 3 interrelated programs. *Sex ki adalat* includes 4 fictional court cases debating about virginity, masturbation, menstruation, and pornography; *Kishor ka shor* offers conversations based on the government-endorsed national adolescent health program RKSK themes featured in *Main Kuch Bhi Kar Sakti Hoon* season 2 regarding obesity, child marriage, and cigarette smoking; *Meri TV duniya* includes trailers and exclusives from *Main Kuch Bhi Kar Sakti Hoon* season 1, 2, and 3. *Khula manch* offers conversations on mental and physical health; and *Purush pariksha* challenges beliefs and pushes viewers to question orthodox ideas.
3. *Kahaniyan sune* (Listen to stories): this option allows the user to engage with specific storylines featuring 7 young female and male characters in *Main Kuch Bhi Kar Sakti Hoon*. These stories include Naveen’s contemplation of adolescent attraction and curiosity and confusion about puberty (*Main young hoon*), Pinky’s story about menstrual hygiene (*Mahine ke vo din*), Shama and Aman’s relationship and their beliefs about consent (*Friendship aur haa*), Sanjay’s issues with peer pressure (*Dosti kya hai?*), Preeta’s survival of an acid attack for being a competent female football player (*Hinsa*), Bunty’s homosexuality (*Attraction aur pyaar*), and Aman’s concerns about bullying (*bullying*).
4. *Games khelein* (Play games or take a quiz): this option allows the user to play quiz games on three themes: *Young sambandh* has questions about adolescent development, *Physical relation* has questions about family planning, and *Log kehte hain* has questions about myths and misconceptions about SRH.
5. Helplines: this option directs users to 2 helplines. One helpline is on SRH through the national toll-free number of the Jansankhya Sthirata Kosh endorsed by the Ministry of Health and Family Welfare. It provides callers with information and counseling on family planning and reproductive health issues, including access and use of contraceptives. The other helpline option links users to a national helpline on gender-based violence for the counseling and reporting of incidents.

In addition to the improved content structure and flow, a critical element of SnehAI (version 2.0) is the use of an NLP system to process and respond to user-entered free-text queries. This NLP system first matches the free texts with prefilled regular expressions (RegEx in short as commonly used by programmers), and if a match is found, corresponding replies are executed. If no matches are found, the NLP system activates a Microsoft Language Understanding (LUIS) conversational AI service and uses it to apply custom machine learning intelligence in client apps (such as AI chatbots, such as SnehAI) to process user input and provide appropriate response [61]. The Azure LUIS app, commonly used in AI chatbots, receives user input in the form of conversational and natural language texts, treats them as *user utterances*, processes the information by extracting *keywords* and predicting *user intentions*, and then uses the JSON response to make decisions about how to fulfill the user’s requests [61].

### Instrumental Case Study

The case study approach is particularly useful for exploring a phenomenon in its natural and real-life context and allows the development of a more in-depth, multifaceted, and holistic understanding [62]. Although this method is well-recognized in disciplines such as business, law, and policy, it needs to be embraced more in global and public health research [63]. We adopted an instrumental case study approach. An *instrumental* (vs *intrinsic* or *collective*) case study focuses on 1 example to gain a broader appreciation of a phenomenon [63,64]. The case in point here is SnehAI as an exemplar of AI chatbots for promoting conversations about SRH. This research method allowed us to explore SnehAI from diverse perspectives, such as technology design, program implementation, and user engagement. We also used a mix of qualitative insights and quantitative data to triangulate our findings.

Qualitative insights were obtained from key stakeholders, with institutional representatives being the coauthors of this paper and others listed in the acknowledgments. A total of 2 in-person group meetings, 4 virtual conferences, and numerous follow-up email discussions took place from September 2018 to December 2020. Half a dozen interim and final reports (including 2 cited in this paper [25,26]), as well as detailed meeting notes, served as foundational documents for our analysis. Quantitative data were obtained through unobtrusive chatbot user behavior tracking with user permission. The chatbot user interactions were mainly captured in 2 ways. First, for any predetermined options indicated in the SnehAI flowchart, the choices were recorded anonymously in a third-party digital analytic tool designed for chatbots on Facebook Messenger, Dashbot [65]. Conversation-specific analytics were aggregated to monitor user engagement and help improve the dynamics of user–bot interactions. Second, a user could also type questions or comments in the dialogue box at any time. These free texts were used as user input in the NLP system to detect user intention and determine the optimal response. For this project, we used the aggregated analytics data from the Population Foundation of India and its partner AI for Good, United Kingdom, gathered from the beginning of May 2020 to the end of September 2020. The data science team at AI for Good, United Kingdom, also randomly selected 15,000 free-text messages with over 20 characters and used machine learning techniques to detect user behavior patterns.

To proactively address potential pitfalls of the case study methodology [63], we adopted a widely accepted and multidisciplinary conceptual framework (ie, the theory of affordances) to guide our inquiries and investigations. We also drew clear boundaries around the specific case (ie, SnehAI 2.0) and the timeframe of user engagement data for our analysis (ie, a 5-month period shortly after the launch). Furthermore, we went through several iterations of data validation, reported discrepancies caused by technical errors, and shared detailed notes with all team members to ensure transparency in the research process.

## Results

### Functional Affordances of SnehAI

To answer research question 1, we compiled an extensive list of relevant affordances reviewed in the literature, evaluated them against the SnehAI app based on the qualitative insights we obtained, and distilled 15 functional affordances (see a summary in Multimedia Appendix 2 [17,33,34,38-41,45,53]). The results presented here specifically address the research gaps we identified. The affordances of a new technology should be primarily about its functionalities. Therefore, these 15 affordances of SnehAI are all based on the functionalities of the chatbot, its AI capacity through the NLP model, the Facebook Messenger mobile app it is embedded in, and the internet and wireless networks that enable its existence. We also included the role of a media personality and relevant legal and social regulatory systems in our assessment. Moreover, we considered both positive and negative sides of each affordance, with the intention of appreciating its actionable possibilities while mitigating potential concerns. Certain labels of these affordances (ie, *accessibility*, *multimodality*, *nonlinearity*, *editability*, and *scalability*) had been previously used in the literature, and some others were differently labeled although they expressed similar ideas (ie, *customizability*, *interactivity*, *visibility*, *glocalizability*, and *connectivity*). Notably, we contributed several new labels (ie, *compellability*, *queriosity*, *trackability*, *inclusivity*, and *actionability*) to represent critical affordances of SnehAI that could apply to similar AI and chatbot apps:

1. *Accessibility* allows SnehAI users to access SRH information that is accurate (with content experts’ approval), trustworthy (as shared by the avatar of a trusted friend), and relatable (with messages expressed in colloquial Hinglish). The subscription option for notifications affords additional access to new information as it becomes available. However, digital inequalities may deprive underserved population groups such as women, youths, and rural residents of smartphone ownership or limit their personal use. Furthermore, users who obtain access to the chatbot can potentially be overwhelmed by new information about taboo topics. It helps that most information on SnehAI is presented as edutainment, both entertaining and educational.
2. *Multimodality* affords SnehAI to be presented through more than one sensory mode to help enhance the user experience. Although common practice in the industry is to simply use plain text in a chat box, SnehAI uses rich media that includes text, audio, and visual modalities, and popular presentation forms, such as GIFs, emojis, and short videos. What SnehAI users see and hear about gender norms may be dramatically different from what is covered in the mainstream media, which could potentially be a source of cognitive dissonance, yet necessary for raising social consciousness about gender inequality [57-59].
3. *Nonlinearity* allows SnehAI to provide curated content through clickable visual menu options instead of a prescribed sequence. In addition, users can enter their queries through free-text messages at any time apart from when they access branched content categories. Thus, SnehAI allows users to take control of their experience as more of a choose-your-own-adventure type of journey. On the flip side, such an adventurous and nonlinear trajectory can also be distracting or confusing for some users who may lose track of a thought as they shift from one content category to another. Currently, there is no separate search bar available in the user interface that allows users to conduct a keyword search from anywhere.
4. *Compellability* allows SnehAI to engage users in compelling ways. The casual, friendly, yet culturally appropriate–looking avatar of SnehAI is rich in visual appeal for Indian adolescents and young adults. Paid Facebook promotions can help attract new users and boost user retention. Furthermore, the triggers and prompts on Facebook Messenger can also attract user attention and provide cues to action. Although no nudges exist presently when a user is inactive for a prolonged time, nudges can be annoying if their intentions are unclear to users [17].
5. *Queriosity* allows SnehAI users to seek answers to their queries based on personal curiosities. For users to be able to enter free-text messages is in stark contrast with the conventional method of preaching to the choir of users. In particular, young people learn better when their genuine curiosity is encouraged without judgment [55,56]. The analysis of the 15,000 sampled queries by our data science team indicated that “SnehAI opened up a safe space for users to ask ‘stupid’ or ‘embarrassing’ questions about SRH without worrying about what others think.” The current version only allows users to query through manually typed text messages. In the future, other query methods may be included. For instance, a search engine may be helpful when the curated content database expands so that users can find and explore the most relevant branched content category. Providing voice input and output capability (as in Siri and Alexa) can be especially useful for users with low levels of literacy.
6. *Editability* allows SnehAI users the ability to craft a message to their satisfaction before sending it out to the chatbot. Thus, users have full control over how they present themselves in this private space. Again, the analysis of 15,000 sampled queries by the data science team showed that most user behaviors were respectful. “For instance, many treated the chatbot as if it was Dr. Sneha, addressing her as Dr, Ma’am ji [respected Ma’am], and didi [older sister].” On the other hand, once a free-text message is sent out, there is no way to revise or recall it, even to correct unintentional mistakes.
7. *Visibility* allows SnehAI to automatically save all messages exchanged between the user and the chatbot and keep them permanently visible in the chat history. Such permanence in visibility can be a convenient information repository over time but also a potential vulnerability, as anyone who has access to the user account can retrieve and review private messages. SnehAI is proud of its strict adherence to the international General Data Protection Regulation on data privacy with a built-in user option to review its detailed privacy policy, including user permission for recording or deleting data.
8. *Interactivity* allows SnehAI to provide immediate feedback on user requests. This allows the chatbot user to simulate real-time interpersonal interaction through turn-taking with verbal expressions and nonverbal cues—through emojis. Therefore, each conversational session can flow like text messaging with a real person. SnehAI is prompt and reliable in responding. However, the free-text message queries by a user are entirely dependent on the quality of the response through the NLP model. When the user intention is incorrectly decoded, the chatbot response is correspondingly off-track. Thus, the quality of conversations with the chatbot is determined by the intelligence of the NLP model in detecting communicative nuances and minimizing bias.
9. *Customizability* allows SnehAI users to interact with curated content and customize their queries based on personal interests, even though there is no option for users to customize their chatbot menus or filter content. Nevertheless, SnehAI uses a hybrid of prefilled RegEx and the LUIS conversational AI client app in its NLP system to customize the chatbot’s response to user queries. In particular, the NLP uses *machine learning features*, such as a phrase list, to detect *user intentions* at various junctions of the user journey [66]. For example, *small talk intents* cover the trivial chit-chat with phrases such as *how are you* and *thank you*. However, a unique aspect of SnehAI is that the NLP model is designed to effectively detect *core intentions* related to topics such as safe sex, reproductive health, and family planning choices. These *core intentions* are identified through keywords that serve as substitutes for *sex*, accounting for the corresponding colloquial expressions by young people and their spellings and misspellings in Hinglish. The NLP system used in SnehAI version 2.0 was initially trained based on insights from the previous version and is being retrained regularly. A part of the LUIS global network, SnehAI contributes Hinglish conversational messages to the larger pool to improve machine learning intelligence over time. It also leverages its growing database for SnehAI NLP training. A more detailed illustration of the LUIS NLP model in SnehAI is provided in Multimedia Appendix 3 within a linked video in Multimedia Appendix 4.
10. *Trackability* allows SnehAI the capability to unobtrusively track certain users’ personal information and interactive behaviors with the chatbot. Because of privacy concerns, Facebook is strict about the kind of personal information recorded in third-party apps. For SnehAI, version 2.0, a formal application was submitted, reviewed, and approved by Facebook to record user’s gender in the Dashbot analytics tracking. Other key performance indicators such as important clickable reactions were also recorded and reported in the *User Engagement With SnehAI* section in *Results*. In addition, inappropriate and abusive user behaviors were also monitored, flagged, and handled to keep SnehAI a safe and civil space. No matter how elaborate the efforts to protect user privacy, some users (particularly those with low digital media literacy) may not fully understand what kind of personal information is being tracked at the back end.
11. *Scalability* allows SnehAI to achieve large-scale user reach by leveraging the popularity and massive user base of Facebook in India, especially among adolescents and young adults. All the information on SnehAI is digitally produced and can be easily replicated for dissemination. As a stand-alone mobile app, SnehAI can be an effective digital tool for frontline health workers to share with young men and women. Harnessing the existing health care and field-based health education networks can easily spread SRH resources on SnehAI across tens of millions of users, with the probability of some creative content going viral. Currently there is no option in SnehAI for users to recommend the chatbot to other Facebook friends. In addition, no aggregate user engagement information is shared in the chat function to further grow the SnehAI user base.
12. *Glocalizability* allows SnehAI to reach the youth living in India and around the globe. The intentional use of colloquial Hinglish makes the AI chatbot particularly approachable and friendly for young people. Regardless of where a user lives, there is a certain level of universality with respect to curiosity, challenges, and experiences related to SRH issues. It is commendable that SnehAI, version 2.0, offers information on 2 nationwide helplines. Unfortunately, no location-based content tailoring is available at this time to contextualize the SRH information for individual users, nor is there a capability to facilitate direct connections with existing local health and social services.
13. *Inclusivity* affords SnehAI to be welcoming of users with diverse backgrounds, especially underserved population groups such as women, youths, and rural residents. Once SnehAI can be accessed, the service is free. In addition, AI chatbots are becoming increasingly capable of processing human voice as user input, such as Siri and Alexa. Rather than depending on the users’ reading and writing skills, a voice-based option can benefit people with low literacy and make SnehAI even more inclusive. Meanwhile, the chatbot presents some privacy risks for users with low digital literacy. Imagine a worrisome parent finding a teenager asking about sexually transmitted infections or a controlling husband finding his wife learning about injectable contraception. With time, other functions may be incorporated to provide additional education and protection to the most vulnerable users.
14. *Connectivity* allows SnehAI meaningful opportunities for parasocial interactions to occur between a user and the chatbot via Dr Sneha’s avatar. Parasocial interactions typically occur in pseudorelationships between a media user and a media personality [67]. For SnehAI users, having someone they can trust to talk about SRH is much better than having no one. According to our data science team, “Many opened up and shared deeply personal issues. Some were keen in knowing more about Dr. Sneha’s whereabouts, wanting to talk in person, and even asked for her WhatsApp number.” In the current version, options are not yet available to safely connect a user with other trusted real-life friends through Facebook Messenger. There is also no direct connection to link one user’s queries with others’ queries to securely facilitate peer-to-peer or social learning.
15. *Actionability* affords SnehAI users to take action as the direct result of their conversations with the chatbot. For example, they can share in-person SRH information that they have learned with their trusted peers. Such private and casual information sharing is, in fact, how many people learn about SRH in the first place. They can also call national helplines to seek professional consultation for themselves or others. Currently, there is no possibility of directly calling through the AI chatbot app or appointment scheduling capabilities with local health or social services. However, with the rapid development of AI technologies such as Google Assistant, more affordances can and will become available for users in due time.

### User Engagement With SnehAI

**Figure 3:**
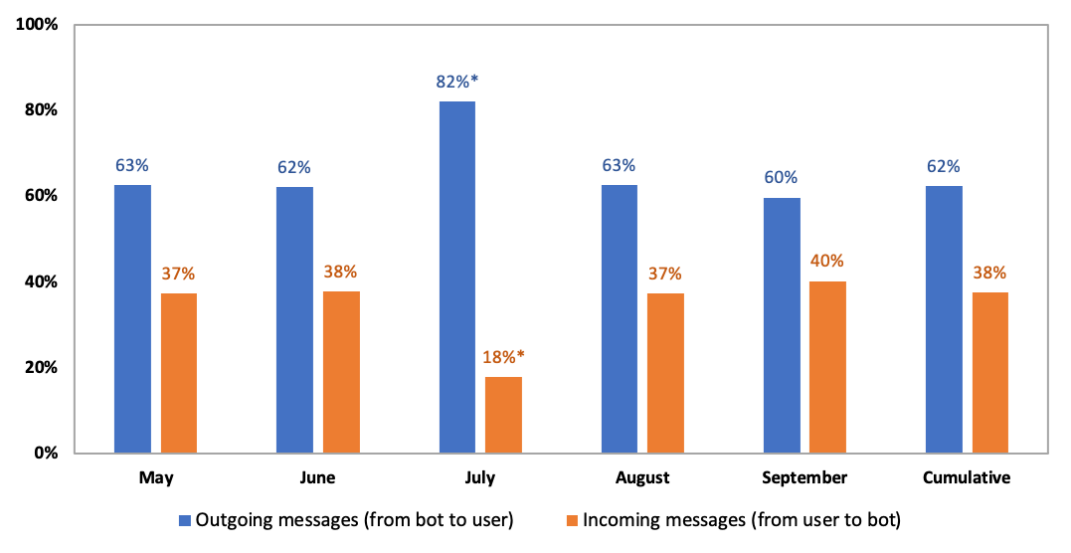
SnehAI chatbot outgoing versus incoming message ratios over time.

To answer research question 2, we examined the aggregate Dashbot analytics data on SnehAI user behaviors. Over a 5-month period, 8,170,879 messages were exchanged between SnehAI and 135,263 unique chatbot users, including 5,100,449 (62.42%) outgoing messages from the chatbot to the users and 3,070,430 (37.58%) incoming messages from the users to the chatbot. The ratios between outgoing and incoming messages were consistently around 60:40 over time, with the exception of repeated outgoing messages in July (such as technical errors, which are marked with asterisks in Figure 3). Specifically, incoming messages from the users to the chatbot were sent through free texts (1,599,339/2,878,908, 52.09%); clickable reactions (1,279,569/2,878,908, 41.67%); or GIFs, audios, images, and videos (191,522/2,878,908, 6.24%).

**Figure 4:**
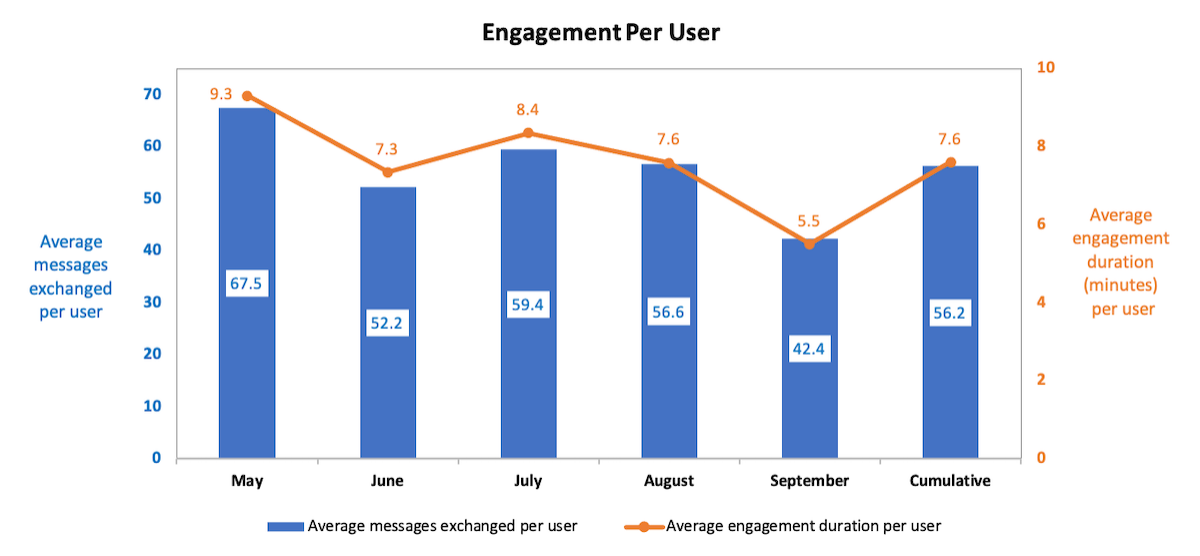
SnehAI chatbot average user engagement over time.

The average user engagement with SnehAI was 1.9 sessions, 7.6 minutes, and 56.2 messages exchanged (Figure 4). The time spent by top users increased from 2 to 3 hours in the first 3 months to 7 hours in 14 sessions in August and 14 hours in 47 sessions in September.

**Figure 5:**
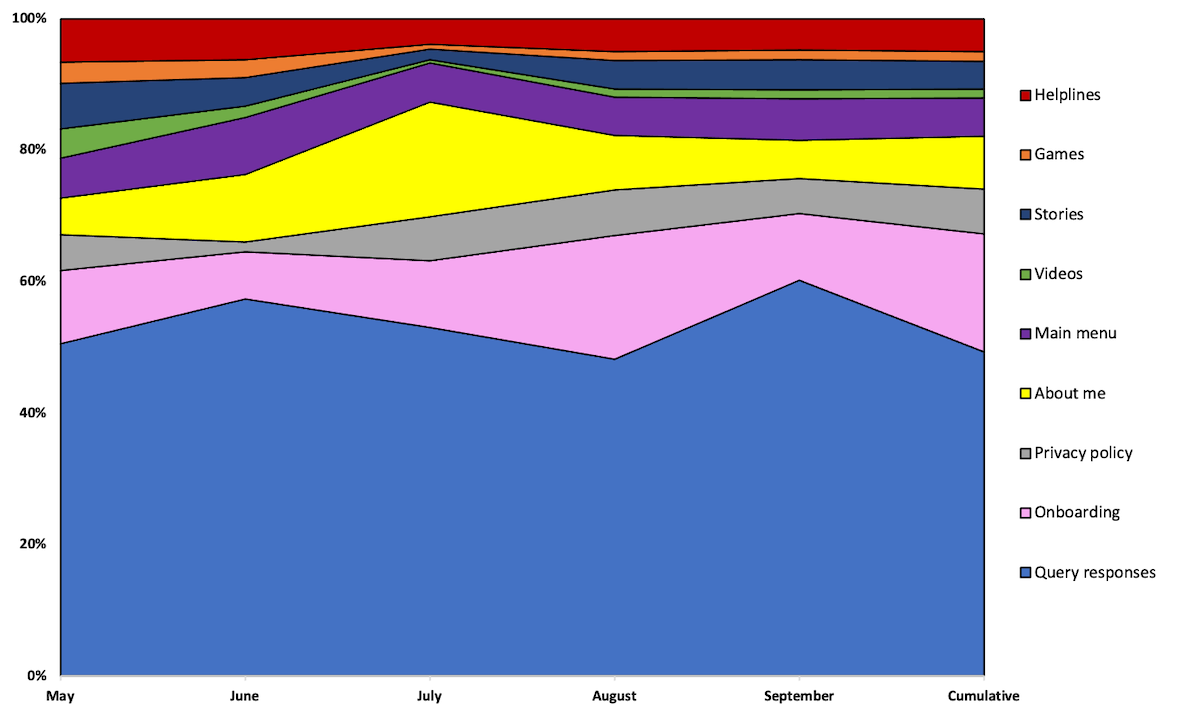
SnehAI chatbot content engagement distribution over time.

User engagement was also tracked through the content categories, including both guided flows using clickable reactions and the handling of free-text messages using NLP. Although multiple messages could have been exchanged with SnehAI each time a user engaged with a particular content category, the overtime trends of engagement frequencies across different content categories provided empirical evidence of user response and preference. From onboarding, to learning about Sneha, to its privacy policy, and its main menu to the videos, stories, games, helplines, and query responses through NLP, the chatbot users traversed across these content areas for 1,430,416 times over the course of 5 months. Approximately half (705,305/1,430,416, 49.31%) of these interactions were about the chatbot responding to the user queries, including small talks and any questions related to themes of health communication (Figure 5). The next highest frequency content category was onboarding (257,042/1,430,416, 17.97%), about SnehAI (115,131/1,430,416, 8.05%), privacy policy (96,305/1,430,416, 6.73%), main menu (83,692/1,430,416, 5.85%), helplines (71,211/1,430,416, 4.98%), stories (61,582/1,430,416, 4.31%), games (20,897/1,430,416, 1.46%), and videos (19,251/1,430,416, 1.35%). These trends in content engagement distribution were relatively consistent (with occasional fluctuations).

**Figure 6:**
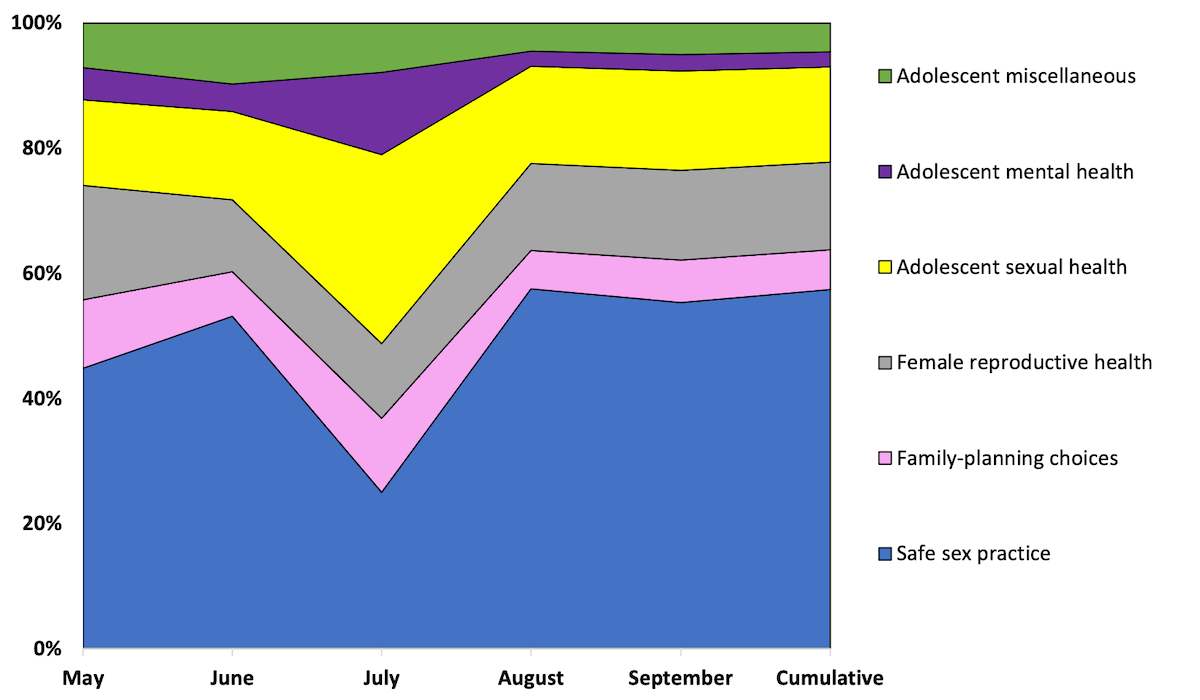
SnehAI chatbot free-text queries content distribution over time.

Our analytics tracking data showed a count of 99,936 typed text messages from the users that SnehAI handled and responded through the NLP system with queries related to the six topical themes (Figure 6): safe sex practices, such as consent, frequency of sexual intercourse, oral and anal sex, impact of other health ailments on sex life, and unplanned pregnancy (57,158/99,936, 57.19%); choice of family planning methods, such as male and female condoms, oral contraceptive pills, intrauterine devices, injectables, and SRH-related themes, such as abortion, sexual intercourse during and after pregnancy, polycystic ovarian disease, and infertility issues (6287/99,936, 6.29%); female reproductive health concerning menstruation (eg, regularity, pain, discharge, and spotting), virginity, and premarital sex (13,965/99,936, 13.97%); adolescent sexual health issues, such as nightfall, masturbation, pornography, sexual stamina, erectile dysfunction, and STIs (15,160/99,936, 15.17%); adolescent mental health issues regarding peer pressure and bullying (2343/99,936, 2.34%); and nutrition and social determinants of health, such as child marriage and gender equality (4623/99,936, 4.63%).

**Figure 7:**
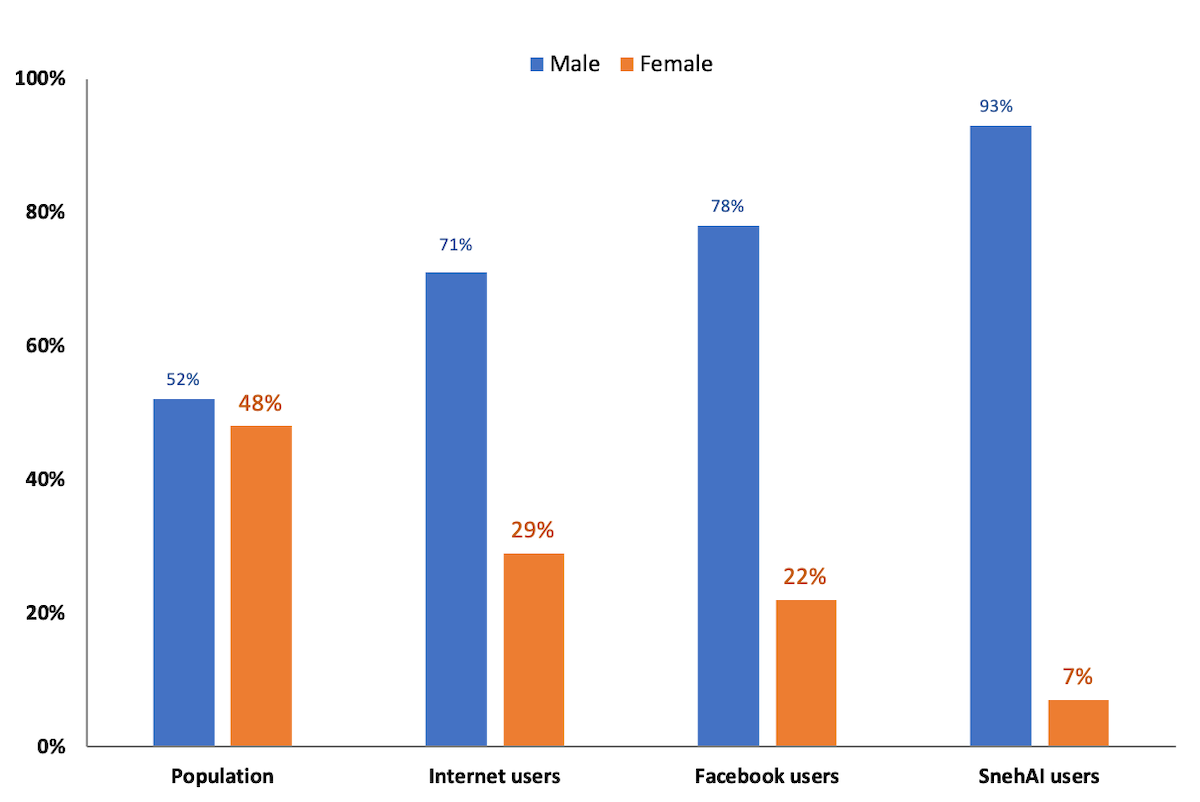
Gender gap in SnehAI users compared with internet and Facebook users in India.

With Facebook’s approval, we accessed the SnehAI chatbot user’s gender-disaggregated data. What we discovered was an extreme gender gap (Figure 7): among the unique chatbot users over this 5-month period, 93% (125,795/135,263) were male, 6.8% (9198/135,263) were female, and 0.2% (270/135,263) unknown. This gender ratio was disproportionally skewed toward male users compared with that in the United Nations Development Programme report on gender distribution of internet users (29% female) and Facebook users (22% female) in India [12], as well as with that of the general population (48% female) according to census data [2].

Even worse, based on the 15,000 sampled queries, our data science team found “a behavioral pattern with female users showing significantly lower self-confidence in their conversations with SnehAI.” This might be a function of female users facing gender disparities in mobile device ownership and low digital media literacy in India.

Furthermore, they are likely less comfortable about openly discussing SRH and may hold higher levels of privacy concerns for digital abuse and sexual exploitation. In contrast, there were incidents where male users requested SnehAI to set up a girl for them, and some even engaged in obscene chats with her expressing intimacy through sexting, asking for porn videos, and using abusive language. A few questions also showed the darkest side of sexual abuse, with male users inquiring which tablets they could give girls if they were unwilling to have sex with them, with the intention of overriding consensual sex.

## Discussion

### Principal Findings

SnehAI pioneered the use of an AI chatbot to engage users, especially young men, in conversations about SRH. A total of 8.2 million messages were exchanged in a 5-month period, with almost half of the incoming user messages being free-text queries comprising personal questions and concerns about SRH. Overall, the SnehAI avatar based on Dr Sneha—the protagonist in *Main Kuch Bhi Kar Sakti Hoon* television show—presented itself as a trustworthy friend and mentor. Furthermore, the curated content from this gender equality and family planning initiative was found to be both entertaining and educational. Moreover, the NLP system worked smoothly and effectively to personalize the chatbot response and optimize user experience.

Using the theory by Gibson to guide our research inquiries and building on the existing literature, we distilled 15 functional affordances of SnehAI. The results of our instrumental case study provide evidence that SnehAI offers a safe space for users to talk about sensitive SRH topics, seek and obtain accurate information, access services locally through helplines, and seek personal counseling:

1. *Accessibility*: SnehAI provided a digital platform for Indian youth to access highly accurate and credible information about SRH in a safe, nonjudgmental space.
2. *Multimodality*: SnehAI used popular media modalities such as videos, GIFs, and emojis to enhance engagement.
3. *Nonlinearity*: SnehAI offered curated content in branched categories that invited nonlinear navigation.
4. *Compellability*: SnehAI compelled the users to open up through her relatable and approachable avatar image and tone.
5. *Queriosity*: SnehAI welcomed queries that encouraged user curiosity about SRH topics.
6. *Editability*: SnehAI enabled users to reword, redraft, and rescript how they showed up in the digital space.
7. *Visibility*: SnehAI enabled users to privately and securely save chat history, making it visible at any point.
8. *Interactivity*: SnehAI simulated conversational turn-taking and immediate verbal and nonverbal feedback.
9. *Customizability*: SnehAI enabled users to navigate the curated content according to their interests and used its machine learning capability to customize responses.
10. *Trackability*: SnehAI tracked users’ web-based behaviors through their clickable actions and self-generated content.
11. *Scalability*: SnehAI holds tremendous potential for scaling up through various adolescent health education programs, networks of frontline health workers, and diverse service providers.
12. *Glocalizability*: SnehAI enabled linkages between young people and service providers in their local areas.
13. *Inclusivity*: SnehAI served as a free digital consultation repository for all users.
14. *Connectivity*: SnehAI created the conditions for users to connect parasocially with a respected and trustworthy avatar at a deeply personal level.
15. *Actionability*: SnehAI used the power of transmedia storytelling to inspire users to take concrete actions to seek help for themselves and their peers.

Meanwhile, we also discovered an extreme gender gap among the many potential disparities. The patriarchal restrictions on women’s autonomy, mobility, and self-expression in India are vividly reflected in SnehAI user analytics data. This disparity in ratio is a risk and can introduce gender bias in the AI chatbot’s NLP model. Our team is currently adapting field-based promotional strategies to include more female users. Other specific opportunities for improvement based on our results include the following:

1. A more intelligent NLP model coupled with targeted behavior change strategies focusing on awareness building and empowerment of women and girls to reduce gender bias.
2. A system setting of reminders and nudges to re-engage idling and inactive users.
3. An option to edit or recall a text message after sending it out to the chatbot or to use voice-based input and output to include users with low literacy.
4. A possibility to customize the menus, filter contents, and search information in the chatbot when the digital artifacts propagate to a larger scale.
5. A feature that allows the user to invite other trusted friends to use the chatbot and leverage anonymous and aggregated user data to boost user participation and peer-to-peer learning.
6. A mechanism to educate the most vulnerable users on how to protect their privacy, safely participate in web-based activities, and advocate for their basic human rights.
7. A network that can directly connect the chatbot users to their local health and social services, for instance, enabling helpline dialing and scheduling appointments through the chatbot.

### Practical Implications

Our findings hold important policy and programmatic implications for health informatics, especially for the design of user-centered and AI-driven interventions. Understanding both positive and negative aspects of the affordances of AI chatbots such as SnehAI can help inform future endeavors to reinforce and amplify the positives and minimize the negatives when designing, implementing, and studying these technologies. The 15 affordances of SnehAI can serve as a comprehensive checklist for similar apps. It helped our research team to pinpoint the unique attributes and strengths of SnehAI and clarify the next steps for future improvements. They can also help chatbot design and analytics teams to select key performance indicators to track over time and monitor user behavioral patterns to ensure the safety of the environment and improve user engagement. This case study also described the NLP model used in SnehAI, version 2.0, to customize the interactions between an AI chatbot and a user. NLP helps optimize the conversation dynamics and personalize the user’s journey, thus getting us closer to accomplishing the United Nations sustainable development goals, focusing on good health and well-being, gender equality, and quality education.

In addition, by covering all relevant aspects of the SnehAI chatbot, we learned that when treating technologies as artifacts, their affordances should include not only the specific app itself. What is equally, if not more, important is to include the information systems and communication networks an app is nested in, as all of them are interconnected and interdependent. SnehAI could not be functional without Facebook Messenger, access to mobile devices, or mobile connections to the internet.

Furthermore, studies on technological affordances should routinely include attention to legal and social regulatory systems that are usually invisible to the public. This is particularly pertinent when a citizenry is divided along the lines of prejudice and hierarchy, such as class, gender, and race. The rapid development of AI technologies will continue to challenge innovators, marketers, and users with moral dilemmas. Our team’s experience working directly with the intended users of SnehAI to respect the circumstances of their living conditions while facilitating meaningful social and behavioral change has demonstrated great value in cocreation and collaboration.

### Theoretical Implications

Our case study on SnehAI demonstrated both the robustness and adaptability of Gibson’s theory of affordances, some 40 years after it was proposed. At the same time, our investigation of SnehAI also sheds light on research gaps in the current literature on technology affordances and offers new insights to expand this theoretical framework. Only when there is a deeper understanding of the design of a user interface and its operating system behind the screen (similar to what our research team did) that it becomes possible to evaluate new technology’s affordances—through their existence, recognition, and realization. Researchers should return to the basic functionalities before jumping to conclusions about other types of social, cognitive, emotional, and therapeutic inferences.

Second, there is a utopian bias in affordances in new media studies. It is important to balance technological determinism and social constructivism when examining the social shaping and consequences of emerging technologies. The identification of affordances by nature is a function of one’s individual capability. An affordance is permanent, but its perception is dependent on the individual’s culture, history, effort, and necessity. Consequently, it is critical for users to be at the front and center of the design. SnehAI was developed keeping the user in mind, as the path of affordances perceived by users in India would be unique to them. Affordances incorporate the microbehaviors exhibited by users, making it imperative that there is a balance between actionable possibilities that are both positive and negative and to proactively address potential risks and concerns, especially when the intention is to better serve the underprivileged populations.

Finally, the human–AI interactions that we observed through SnehAI were intriguing. This can be viewed as a contemporary version of what Horton and Wohl [67] called *parasocial interaction* between mass media users and media personalities on radio or television. It can also be viewed as a variation of *player–avatar interaction* in game research [68]. The narrative connection to SnehAI and its friendly avatar in a private conversational setting offers several opportunities for theoretical development in human–computer interaction and computer-mediated communication.

### Limitations and Future Research

Our instrumental case study did not include any primary data collection from SnehAI users. This is a necessary next step. Although *Main Kuch Bhi Kar Sakti Hoon* Facebook page analytics provided useful proxies for Facebook users, direct contact with the AI chatbot users themselves using surveys would be immensely useful to pursue. With their permission, such self-reports can gain invaluable knowledge about SnehAI users’ characteristics, such as age, gender, geographic location, and socioeconomic status. In particular, this user profiling approach can provide empirical evidence about several affordances discussed in this paper, namely, accessibility, inclusivity, glocalizability, and scalability.

Additional theoretical models of technology acceptance [69], uses, and gratifications [70] may further deepen our understanding of key factors that help individuals decide to adopt chatbots such as SnehAI, the specific cognitive, affective, and social needs that are fulfilled, and reasons why certain users would choose to return and continue their conversations with the chatbot repeatedly. These aspects of user motivations and behaviors can be assessed through cross-sectional or longitudinal surveys and triangulated with in-depth interviews. Comparing different groups, such as nonusers, light users, and heavy users, can provide additional insights into psychological and contextual determinants.

Although SnehAI, version 2.0, is a stand-alone app, the avatar is still based on the protagonist of *Main Kuch Bhi Kar Sakti Hoon*, and much of its user content is still curated from 3 seasons of the popular television serial drama—183 episodes, 30 minutes in duration and transmedia extensions on digital platforms. This connection gave SnehAI chatbot a compelling face or interface and connected it to a much larger background story with rich SRH information and effective behavior modeling [56,57]. To better study the social impact of this AI chatbot, a field- and web-based experiment can be conducted to test the differences between SnehAI as a stand-alone SRH intervention, SnehAI as a transmedia edutainment extension, and conventional SRH education without SnehAI among Indian adolescents and young adults that need it the most.

### Conclusions

As the first *Hinglish* (Hindi and English) AI chatbot deliberately designed for social and behavior change communication, SnehAI is an innovative, unique, and promising app for engaging vulnerable and hard-to-reach populations groups in the context of SRH education and discussion. It offered a private, nonjudgmental, and safe space for users to talk about otherwise sensitive topics, obtain accurate and trustworthy information, access national services through toll-free helplines, and seek personalized consultations. It also opened up exciting opportunities to leverage the emotional appeal of storytelling through thoughtful yet entertaining content, positive outlook of an avatar, relatable verbal and nonverbal expressions, and friendly tone of voice to effectively engage young people in talking and learning about SRH. The comprehensive checklist of affordances and critical user engagement analytics from this case study are not only a powerful testimonial of SnehAI itself but also a significant representation of the potential and impact of AI technologies on social good.

## Appendix

Multimedia Appendix 1 *Main Kuch Bhi Kar Sakti Hoon* Facebook page user insights.

Multimedia Appendix 2 The functional affordances of SnehAI chatbot.

Multimedia Appendix 3 An illustration of Language Understanding (LUIS) natural language processing system in SnehAI.

Multimedia Appendix 4 Video illustration of Language Understanding (LUIS) natural language processing system in SnehAI.

## Abbreviations

AI: artificial intelligence
GIF: graphics interchange format
LUIS: Language Understanding
NLP: natural language processing
RKSK: Rashtriya Kishor Swasthya Karyakram
SRH: sexual and reproductive health
