# Supplementary material for: An Artificial Intelligence Chatbot for Young People’s Sexual and Reproductive Health in India (SnehAI): Instrumental Case Study
Source: J Med Internet Res. 2022 Jan 3;24(1):e29969. doi: 10.2196/29969 (PMC8764609; doi:10.2196/29969)
Supplement: Multimedia Appendix 1 [file jmir_v24i1e29969_app1.docx]

**Multimedia Appendix 1**. *Main Kuch Bhi Kar Sakti Hoon* Facebook Page User Insights


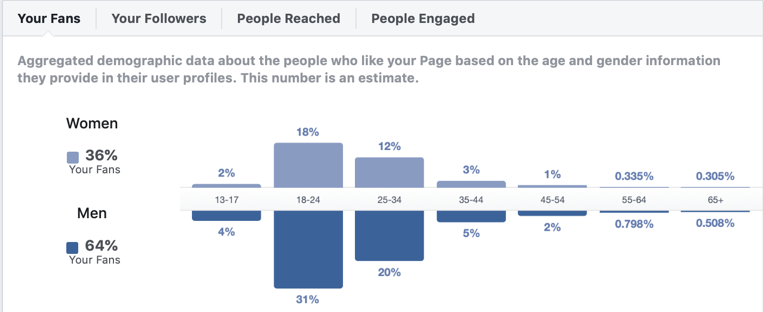

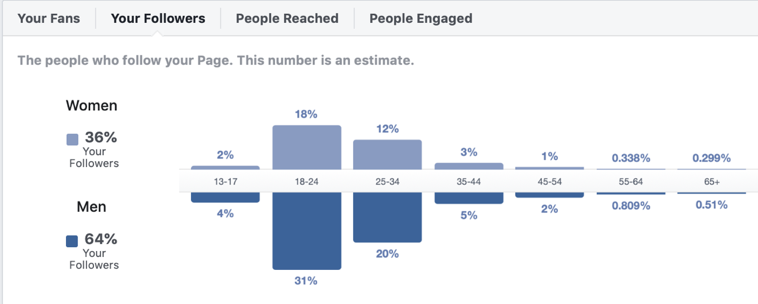


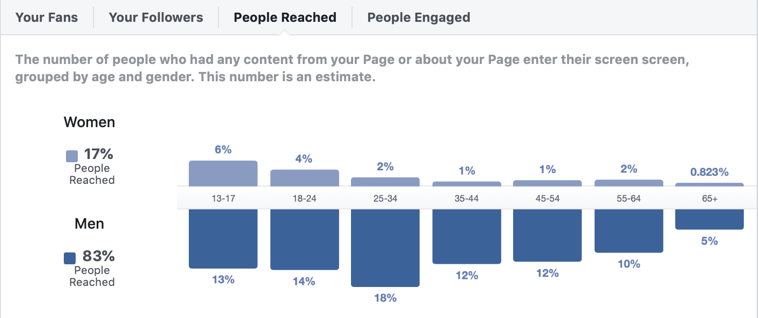

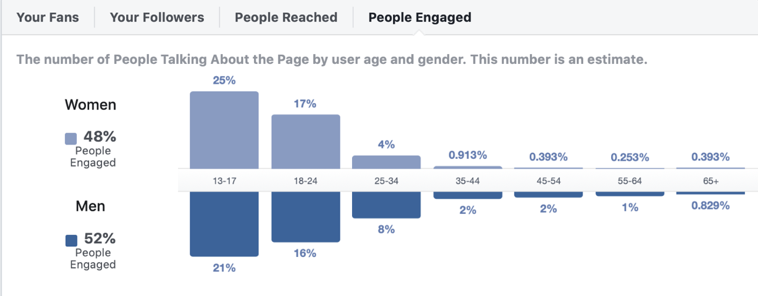


In October 2019, Facebook Insights Analytics showed that, on the *Main Kuch Bhi Kar Sakti Hoon* Faceboook Page, there was an accumulation of 133,052 fans who clicked the “like” button, 134,423 users who clicked the “follow” button, 257,205 users reached, and 7,102 users engaged.  Based on their profile information, the fans and followers were mostly 18 to 34 years old (81%), the most engaged users were 13-24 years old (79%); men were more active than women (64% of fans, 64% of followers, 83% users reached, and 52% users engaged), and a majority of these active users were living in cities such as Delhi, Mumbai, Kolkata, Lucknow, Ahmedabad, Patna, Kanpur, and Bangalore, according to Facebook Insights estimates.
