# Supplementary material for: An Artificial Intelligence Chatbot for Young People’s Sexual and Reproductive Health in India (SnehAI): Instrumental Case Study
Source: J Med Internet Res. 2022 Jan 3;24(1):e29969. doi: 10.2196/29969 (PMC8764609; doi:10.2196/29969)
Supplement: Multimedia Appendix 2 [file jmir_v24i1e29969_app2.docx]

**Multimedia Appendix 2.** The Functional Affordances of SnehAI Chatbot

| **Affordances** | **Positives / Action Possibilities** | **Negatives / Potential Concerns** | **Related References** |
| --- | --- | --- | --- |
| 1. *Accessibility* | users can access information that is accurate, trustworthy, prompt, and relatable; subscription for notifications | digital inequality for the underprivileged such as women, youth, and rural villagers; information overload | [38-40] |
| 2. *Multimodality* | messages from the chatbot are presented through multiple sensory modes such as text, audio, visual, and a combination | confusion with commonly sensitized mainstream media coverage in compliance with regressive gender norms | [17, 38,40] |
| 3. *Nonlinearity* | content presented via clickable visual menus of options for a personalized user journey and free text queries any time | easy to lose the track of the location of specific content especially for users with low media literacy; no separate search bar available as part of the user interface | [17,38,41] |
| 4. *Compellability* | Facebook paid promotion; friendly avatar; triggers and prompts on Facebook Messenger for user engagement | can be distracting or annoying if the intention and value are not clearly conveyed to the user | [17] |
| 5. *Queriosity* | search for answers to queries based on personal curiosities by entering free text messages to the chatbot | no search engine available to find relevant content in the chatbot database; no voice input and output for user queries | [17,41] |
| 6. *Editability* | users can take the time to craft a message before sending it out; users can control their self-presentation in a private space | once the free text messages are sent to the chatbot, there is no way to revise or recall the content in the chat box | [38, 45] |
| 7. *Visibility* | all messages are automatically saved and permanently visible in the private chat history; transparent with privacy policy | anyone who has access to the user account can retrieve and review the messages exchanged through the private chat history | [41,45] |
| 8. *Interactivity* | immediate feedback upon user request; real-time interactions; conversations flow like text messaging with a real person | the free text message queries are entirely dependent on the quality response through the LUIS natural language processing app | [17,38] |
| 9. *Customizability* | user queries are customized based on individual interests; branched content consumption is based on personal choices | no option for users to customize their chatbot menus or filter the existing content by information source | [39,45] |
| 10. *Trackability* | certain user personal information and interactive behaviors with the chatbot are tracked unobtrusively by the system | Users with low digital media literacy may not fully understand what information of theirs is being tracked behind the screen | [38,39,53] |
| 11. *Scalability* | Facebook platform for large-scale user reach, especially youth; digital content can be easily replicated for viral spread | no option to recommend the chatbot to other Facebook friends yet; no aggregated user engagement info shared through chat | [40,41,45] |
| 12. *Glocalizability* | colloquial Hinglish that can reach Indians living in the country and also around the globe; national helplines available | no location-based content tailoring such as local health and social service resources to contextualize user information | [38,39,40] |
| 13. *Inclusivity* | inclusive of diverse and underserved population groups such as women, youth, and rural villagers; free service to all | potential risks for underprivileged users such as women and youth with low digital media literacy to protect their privacy | [39,40,53] |
| 14. *Connectivity* | connections may be established through parasocial interactions between a user and the chatbot via Sneha’s avatar | no direct connections are linked between a user’s queries and their peers’ and no options to safely connect other friends | [39,40,45] |
| 15. *Actionability* | national helpline numbers are available for interested users to take action and seek help for themselves or loved ones | no direct calling the helplines through the chatbot app or making appointments with local health or social services available | [30,33,34] |
