# Supplementary material for: An Artificial Intelligence Chatbot for Young People’s Sexual and Reproductive Health in India (SnehAI): Instrumental Case Study
Source: J Med Internet Res. 2022 Jan 3;24(1):e29969. doi: 10.2196/29969 (PMC8764609; doi:10.2196/29969)
Supplement: Multimedia Appendix 3 [file jmir_v24i1e29969_app3.docx]

**Multimedia Appendix 3**. An Illustration of LUIS Natural Language Processing System in SnehAI

Based on the insights from user conversations in SnehAI version 1.0, thematic *user intents* were designed in the natural language processing system in SnehAI version 2.0 for handling conversations related to sexual and reproductive health. These thematic intents are trained through *user utterances* for subjects covered by matching *keywords*. Below is an example of the *machine-learning feature* phrase lists (including substitutes, colloquial expressions, and misspellings) and 12 sample training sentences for keyword “sex.”


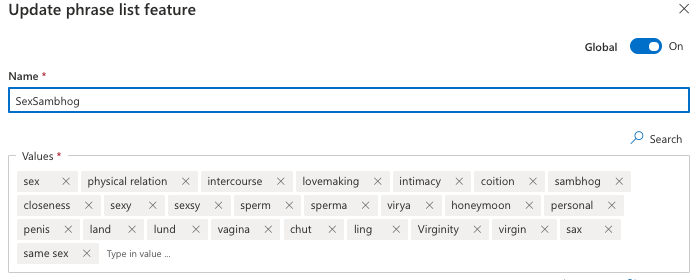


Sample training sentences for keyword “sex”:

| 1. *sex ki bimaari ke baare me batao* | *Want to learn about sex related diseases* | *7. mujhe baccha nahi chahiye* | *I don’t want the child* |
| --- | --- | --- | --- |
| 1. *baccha girana hai* | *Have to abort the child* | *8. meri biwi pregnant hai* | *My wife is pregnant* |
| 1. *abortion kaise hota hai* | *How does abortion work* | *9. STD kya hote hai* | *What are STDs* |
| 1. *mujhe pregnant nahi hona* | *I don’t want to be pregnant* | *10. sex beemari se kaise bache* | *Want to learn about sex related diseases* |
| 1. *sex karna hai* | *Want to have sex* | *11. sex kaise karte hai* | *How to have sex* |
| 1. *meri girlfriend ko sex nahi karna* | *My girlfriend doesn’t want to have sex* | *12. STD se kaise bache* | *How to avoid STDs* |

When a user sends a text message which falls in below categories, the chatbot maps the message to respective user intents and responds with customized options in a carousel, featuring multiple graphic covers with linked rich content. For example, if a user sends a typed message saying “with condom, it’s not fun”, the chatbot will identify the keyword “condom” and its corresponding use intent of “family planning choices”, then respond in a carousel within linked stories, games, quizzes, and helpline (see [attached video](https://www.dropbox.com/s/uy85yicg06m46o7/%20SnehAI_MultimediaAppendixB.mp4?dl=0)). This illustrates how the NLP personalizes user experience according to individual queries with different rich content options that a user can choose from.

| **User Utterance Examples (*in Hinglish + English translation*)** | | **Sample Topics** | **Themes of User Intents** | **Custom Response Carousels** | | | | |
| --- | --- | --- | --- | --- | --- | --- | --- | --- |
| *meri girlfriend ko sex nahi karna* | *My girlfriend doesn’t want to have sex* | Consensual sex | Safe sex strategies |  | Story: Attraction aur Pyaar; Friendship aur Haa, Hinsa |  | Quiz: Young, PatiPatni, LogKheteHain | Helpline: Jansankhya Sthirata Kosh |
| *sex ki bimaari ke baare me batao* | *Want to learn about sex related diseases* | STD |  |  |  |  |  |  |
| *baccha girana hai* | *Have to abort the child* | Abortion |  |  |  |  |  |  |
| *mujhe baccha nahi chahiye* | *I don’t want the child* | Unplanned pregnancy |  |  |  |  |  |  |
| *Girlfriend pregnant ho gayi* | *Girlfriend is pregnant* | Teenage pregnancy |  |  |  |  |  |  |
| *mujhe pregnant nahi hona* | *I don’t want to be pregnant* | Don’t want to get pregnant |  |  |  |  |  |  |
| *sex karna hai* | *Want to have sex* | Sex |  |  |  |  |  |  |
| *DMPA kya hai* | *What is DMPA* | DMPA | Family planning choices |  | Story: Dosti Kya Hai; Attraction Aur Pyaar; Friendship Aur Haa | Games: Log Kehte Hai | Quiz: Physical relation | Helpline: Jansankhya Sthirata Kosh |
| *Condom me maja nahi aata* | *With condom, it’s not fun* | Condom |  |  |  |  |  |  |
| *I pill kaise lete hai* | *How to take the pill* | Contraceptive pills |  |  |  |  |  |  |
| *Copper T kyun use karte hai* | *What's the use of Copper T* | Copper T |  |  |  |  |  |  |
| *virgin kaise pata kare* | *How to find out if she is a virgin* | Virginity | Female reproductive health | Video: Sex ki Adalat-Menstruation, Sex ki Adalat-Virginity | Story: Periods | Games: Log Kehte Hai | Quiz: Physical relation |  |
| *periods me dard hota hai* | *I have pain during periods* | Menstruation |  |  |  |  |  |  |
| *ruk ruk kar period hota hai* | *Periods are not consistent* | Spotting |  |  |  |  |  |  |
| *white discharge hota hai* | *There is white discharge* | Discharge |  |  |  |  |  |  |
| *night fall kyun hota hai* | *What's the cause for nightfall* | Nightfall | Adolescent sexual health | Video: Sex ki Adalat-Masturbation, Sex ki Adalat-Pornography | Story: Dosti Kya Hai; Attraction aur Pyaar |  | Quiz: Young Sambandh |  |
| *mujhe hast maithun rokna hai* | *I want to stop masturbating* | Masturbation |  |  |  |  |  |  |
| *Porn video dekhna hai* | *Want to watch porn* | Pornography |  |  |  |  |  |  |
| *Main gay hu* | *I am gay* | Same sex |  |  |  |  |  |  |
| *Dost galat kaam karte hai* | *Friends are involved with inappropriate stuff* | Peer pressure | Adolescent mental health | Video: Khula Manch, Sex ki Adalat, Kishor ka shor, Purush Pariksha | Story: Bullying Dosti Kya Hai? | Games: Log Kehte Hai | Quiz: Young Sambandh |  |
| *Mere dost mujhe chedte hai* | *My friends tease me* | Bullying |  |  |  |  |  |  |
| *Bohat pressure me hun* | *I am in a lot of pressure* | Mental health |  |  |  |  |  |  |
| *kya bal vivaah theek hai* | *Is child marriage right?* | Child marriage | Adolescent miscellaneous | Video: Kishor ka shor-sehat ka khayal; Kishor ka shor-bal vivah; Purush Pariksha; | Story: Friendship aur Haa; Hinsa |  |  | Helpline: Gender-based violence |
| *kya ladka ladki barabar hai* | *Are boys and girls equal?* | Gender equality |  |  |  |  |  |  |
| *biwi kaam karna chahti hai* | *Wife wants to work* | Women empowerment |  |  |  |  |  |  |
| *acchi sehat banani hai* | *Want to be healthy* | Nutrition |  |  |  |  |  |  |
